# Reoperation for Pyriform Sinus Fistula in Pediatric Patients

**DOI:** 10.3389/fped.2020.00116

**Published:** 2020-04-03

**Authors:** Qingfeng Sheng, Zhibao Lv, Weijue Xu, Jiangbin Liu

**Affiliations:** Department of General Surgery, Shanghai Children's Hospital, Shanghai Jiao Tong University, Shanghai, China

**Keywords:** pyriform sinus fistula, reoperation, recurrence, children, endoscopy

## Abstract

**Introduction:** The aim of this study was to analyze the authors' experience in re-operative surgery for children with pyriform sinus fistula (PSF) who were subjected to attempted but failed operations.

**Methods:** We retrospectively analyzed the medical records of 30 patients with PSF who underwent reoperation (i.e., a revision of the primary performed definitive procedure) from January 2010 to December 2018.

**Results:** There were 19 boys and 11 girls. Twenty-nine cases were left-sided. The median age of the patients when they underwent the primary operation was 5.5 years (range, 15 days−14 years). Five children received two definitive procedures from outside hospitals. The primary operations included traditional open-neck surgery (*n* = 30), endoscopic-assisted open-neck surgery (*n* = 4), and endoscopic laser cauterization (*n* = 1). The median time from primary operation to recurrence was 4 months (range, 1 month−4 years). The reasons for recurrence were incomplete resolution of infection (*n* = 7), incomplete resection of the fistula (*n* = 23), cauterization of PSF inner orifice (*n* = 1), only cyst excision in neonates (*n* = 2), and unknown (*n* = 2). All 30 children underwent endoscopy-assisted open-neck surgery. The median age of the children when they underwent reoperation was 8 years (range, 2–17 years). The fistula was detected in 29 cases (96.7%). After reoperation, good outcome was achieved in 27 patients (90%). Wound infection developed in one case. PSF recurred in two cases (6.7%).

**Conclusion:** Most of the recurrences observed by us are preventable. Complete resolution of infection, clear verification, and exact resection of the fistula at a high level are essential for preventing recurrence. Endoscopy-assisted surgery is effective for PSF reoperation.

## Introduction

Pyriform sinus fistula (PSF) is a rare clinical entity in pediatric patients (1–3). Management options described in the literature have included traditional open-neck surgery, endoscopy-assisted open-neck surgery, and endoscopic obliteration of the sinus tract alone (using coblation or electro-, chemo-, or laser cauterization), with a reported recurrence rate of 1.4–44.1% (3–8). The aim of this study was to analyze the authors' experience in reoperative surgery for children with PSF who were subjected to attempted but failed operations.

## Patients and Methods

The authors have operated on 205 children of PSF between January 2010 and December 2018. From this, 175 were primary and 30 were reoperative cases. Reoperation was defined as a revision of the primary definitive procedure. The exclusion criteria are as follows: patients who had not yet undergone definitive surgery after recurrence, who underwent incision and drainage (I&D) only, who had incomplete data, withdrawal of treatment. The medical records of 30 patients who underwent reoperation were analyzed retrospectively. Twenty-seven children received primary surgery from outside hospitals. Special emphasis was placed on the analysis of the operative notes of the original procedures. The details of endoscopy-assisted open surgery have been described previously by our group (3, 5). Follow-up was conducted by phone or interview in our clinics (Q. Sheng). Ethical approval was obtained from the Ethics Boards of Shanghai Children's Hospital (#2019R017). Written informed consents were obtained from parents and/or legal guardians on behalf of the children in accordance with the Declaration of Helsinki.

## Results

In this series, there were 19 boys and 11 girls. Twenty-nine cases were left-sided, and one case was right-sided. The median age of the patients when they underwent the primary operation was 5.5 years (range, 15 days−14 years), and two patients were operated on in the neonatal period. Five children received two definitive procedures from outside hospitals. The primary operations included traditional open-neck surgery (*n* = 30), endoscopic-assisted open-neck surgery (*n* = 4), and endoscopic laser cauterization (*n* = 1). Previous treatments before first definitive operation were I&D in 18 cases (the range of I&D attempts, 1–9) and conservative therapy in 12 cases.

**Figure 1:**
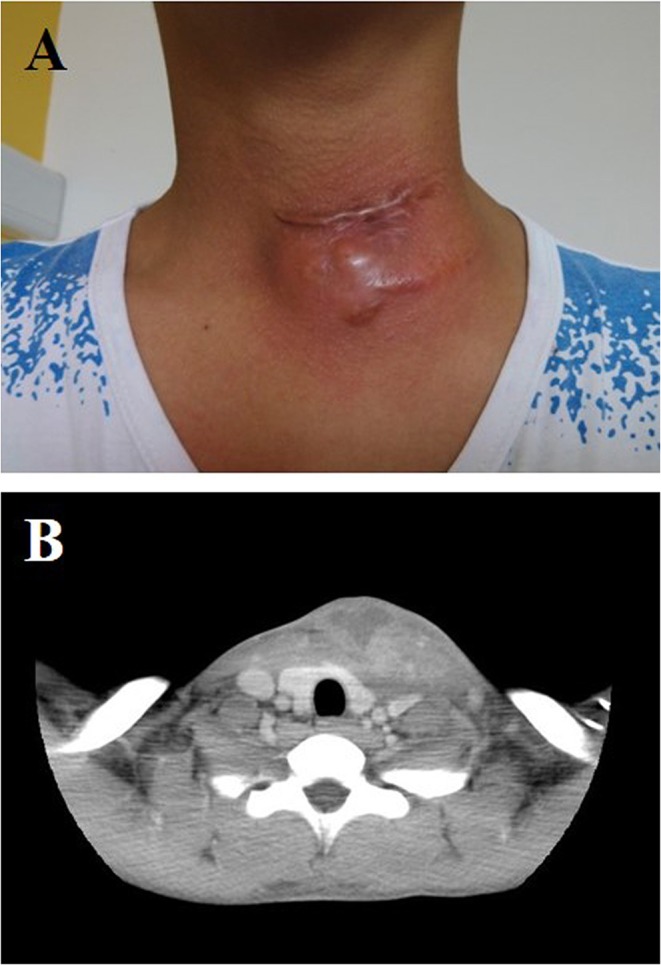
**(A)** Neck abscess formed 2 years after the primary operation in an 11-years-old boy (written informed consent was obtained from the patient and his parent for the publication of this image). **(B)** CT axial scan showed abscess in the left side of the neck.

**Figure 2:**
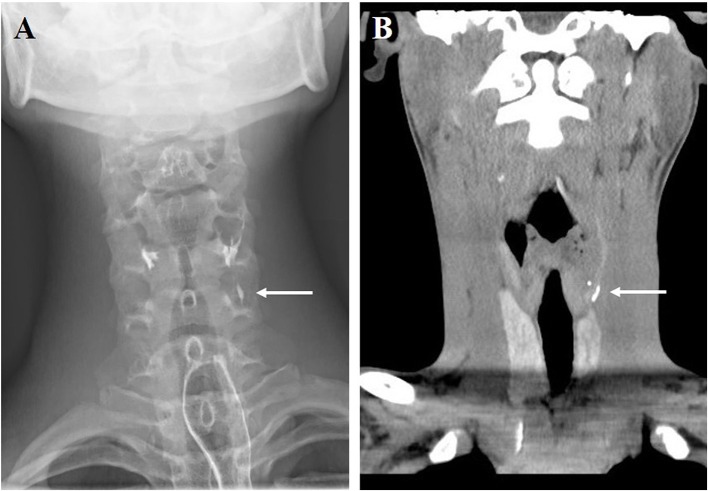
Barium esophagography **(A)** and CT coronal scan **(B)** showed pyriform sinus fistula in non-infected state (arrow, the same patient in Figure 1).

**Figure 3:**
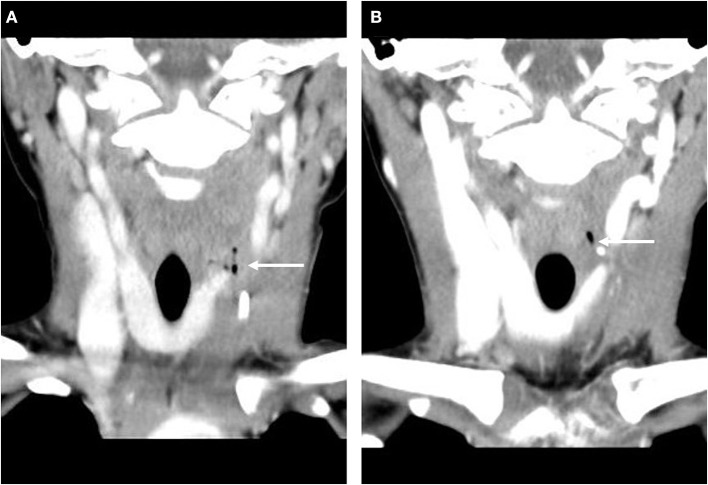
CT coronal scan showed air density before primary operation (**A**, arrow) and reoperation (**B**, arrow).

All recurrent cases presented with neck infection or abscess (Figure 1). The recurrence was confirmed by a barium esophagography and CT scan, showing the fistula tract in the non-infected state (Figures 2, 3). The median time from definitive procedure to recurrence was 4 months (range, 1 month−4 years). Treatments after recurrence were I&D in 22 cases (median, 2; range, 1–8) and antibiotics therapy in eight cases.

The reasons for recurrence were incomplete resolution of infection (*n* = 7), incomplete resection of the fistula (*n* = 23), cauterization of PSF inner orifice (*n* = 1), only cyst excision in neonates (*n* = 2), and unknown (*n* = 2).

Seven patients underwent primary operation before resolution of acute infection, with an average time interval of 1.2 months. The conclusion of incomplete resolution of infection was drawn by analysis of the operative notes of the original procedures. It is hard to find the fistula tract in the infected state even with the help of endoscopy.

The operative notes of 23 patients demonstrated identification but incomplete excision of the fistula. Most likely the surgeons were trying to mobilize and resect the fistula entirely. However, partial resection occurred because of fibrosis and scar formation around the tract, rupture of the fistula during the exploration, unawareness of the variations in the course of the fistulas, or technical errors.

**Figure 4:**
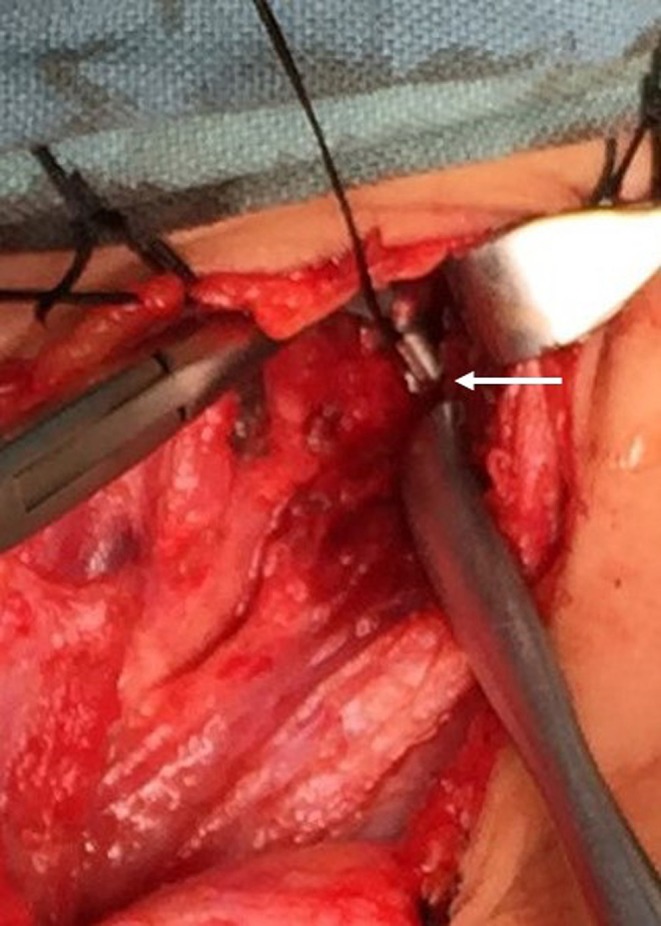
Operative view of the remaining fistula tract (arrow).

One patient received endoscopic laser cauterization of the inner opening, but PSF recurred 1 month later. Two patients underwent cyst excision alone in the neonatal period. These two newborns were misdiagnosed as lymphangioma and simple neck cyst, respectively. Letting the fistula tract remain resulted in recurrence (Figure 4).

All 30 children underwent endoscopy-assisted open-neck surgery. The median age of the children when they underwent reoperation in our hospital was 8 years (range, 2–17 years). Reoperation was performed after the resolution of acute infection (median, 3 months). The fistula was detected successfully with intraoperative endoscopy in 29 cases (96.7%) but failed in one case due to severe fibrosis. The median follow-up period of this series was 3 years (range, 8 months−7 years). After reoperation, good outcome was achieved in 27 patients (90%). Wound infection developed in one case and was managed by changing the dressing. PSF recurred again in two cases (6.7%), with 3 months and 1 year after reoperation, respectively.

## Discussion

PSF represents only 3–10% of all branchial malformations. The classic presentations are neck abscess, acute suppurative thyroiditis, neck mass with or without dyspnea, and thyroid lesion. Treatment for PSF has historically been complete surgical resection of the entire fistula tract. We and other investigators have reported that intraoperative endoscopy could facilitate identification of the fistula, resulting in a low rate of recurrence (3–5, 9–11). Recently, endoscopic obliteration of the sinus tract, using different modalities (e.g., coblation or electro-, chemo-, or laser cauterization), has shown successful outcomes (6–8, 12–16). However, the risk of recurrence was relatively high (8.7–44.1%).

Complications after treatment of PSF include recurrence, wound infection, vocal cord motion impairment/vocal cord paralysis, salivary fistula, Horner syndrome, injury to the recurrent or superior laryngeal nerves, and facial nerve paralysis. Most of the complications can be treated conservatively. Patients with recurrence need definitive reoperative procedures. The reoperative cases in our series was high (14.6%), as ours is a referral center for the management of branchial malformations in children.

The most common reason for recurrence in this series was incomplete resection of the fistula. Identification of the fistula within the fibrotic tissue after repeated infection and I&D can be challenging even for experienced surgeons. Insertion of a catheter or injection of blue dye into the fistula with the help of endoscopy was reported to be very useful to find the tract. Improper high pressure during dye injection could result in rupture of the fistula tract. Special emphasis should be placed on the anatomy variations in the course of the fistula. Xiao et al. (4) reported three types of fistula courses according to their anatomic relationship with the inferior cornu of the thyroid cartilage (ICTC). Exposure of the ICTC might simplify dissection of the proximal part of the fistula. The tract was ligated and resected near the apex of the pyriform fossa. *En bloc* resection of the fistula, surrounding fibrotic tissue, and a portion of the thyroid was indicated in some patients.

A number of patients with PSF suffered from several episodes of infection. Turning to the issue of optimal timing for surgical intervention, definitive treatment should be considered 8–12 weeks after resolution of acute infection. Patients might exhibit gradual clinical recovery and laboratory normalization after the inflammatory episode. Pediatric surgeons could still find infectious and swollen soft tissues in the operative field and hard to detect the fistula tract. Operation performed too early always results in failure, which is unacceptable.

Obliteration of the inner orifice is supposed to be an effective, quick, and simple way for the management of PSF. Moreover, this procedure can be performed even in the infected state. However, recurrence is inevitable in some patients due to the remaining of the fistula tract. Wang et al. (7) reported that the success rate was 55.9% after first laser cauterization; 85.2% after two treatments. Cha et al. (8) reported that the success rate after the first trichloroacetic acid (TCA) chemocauterization was 77.3%. It seems reasonable to perform complete resection of the fistula when multiple recurrences occur.

Correct diagnosis preoperatively in the neonatal period is vital to minimize the possibility of recurrence. We and other groups reported that CT scan was superior to barium esophagography for newborns when PSF is suspected (3, 17–20). Total cyst removal with fistula tract excision is the suitable definitive treatment for neonatal PSF.

As for the procedure for reoperation, endoscopy-assisted open-neck surgery was conducted in our series with a recurrence rate of 6.7%. Wang et al. (7), Cha et al. (8), and Yanagisawa et al. (14) reported that endoscopic obliteration of the sinus tract could be performed for recurrent cases, with a successful rate of 66.7–70%.

There are several limitations of this study: retrospective design, small number of reoperative cases, lacking long-term follow-up.

In conclusion, we believe that most of the recurrences observed by us are preventable. Correct diagnosis is essential to avoid inadequate operation for newborns and children (21). The optimal timing for surgical intervention was 8–12 weeks after resolution of acute inflammation. Resection of the entire fistula contributes significantly to good outcomes. Moreover, endoscopy-assisted open-neck surgery is effective to treat recurrent patients.

## Data Availability Statement

All datasets generated for this study are included in the article/supplementary material.

## Ethics Statement

The studies involving human participants were reviewed and approved by Ethics Boards of Shanghai Children's Hospital. Written informed consent to participate in this study was provided by the participants' legal guardian/next of kin.

## Author Contributions

QS, ZL, WX, and JL collected and analyzed the clinical data. QS and ZL designed the experiment and wrote the manuscript. All authors have read and approved the final manuscript.

### Conflict of Interest

The authors declare that the research was conducted in the absence of any commercial or financial relationships that could be construed as a potential conflict of interest.
